# High Salt Intake Down-Regulates Colonic Mineralocorticoid Receptors, Epithelial Sodium Channels and 11β-Hydroxysteroid Dehydrogenase Type 2

**DOI:** 10.1371/journal.pone.0037898

**Published:** 2012-05-31

**Authors:** Daniel Lienhard, Meret Lauterburg, Geneviève Escher, Felix J. Frey, Brigitte M. Frey

**Affiliations:** 1 Department of Nephrology and Hypertension, University Hospital of Berne, Berne, Switzerland; 2 Department of Clinical Research, University Hospital of Berne, Berne, Switzerland; Emory University, United States of America

## Abstract

Besides the kidneys, the gastrointestinal tract is the principal organ responsible for sodium homeostasis. For sodium transport across the cell membranes the epithelial sodium channel (ENaC) is of pivotal relevance. The ENaC is mainly regulated by mineralocorticoid receptor mediated actions. The MR activation by endogenous 11β-hydroxy-glucocorticoids is modulated by the 11β-hydroxysteroid dehydrogenase type 2 (11β-HSD2). Here we present evidence for intestinal segment specific 11β-HSD2 expression and hypothesize that a high salt intake and/or uninephrectomy (UNX) affects colonic 11β-HSD2, MR and ENaC expression. The 11β-HSD2 activity was measured by means of 3H-corticosterone conversion into 3H-11-dehydrocorticosterone in Sprague Dawley rats on a normal and high salt diet. The activity increased steadily from the ileum to the distal colon by a factor of about 3, an observation in line with the relevance of the distal colon for sodium handling. High salt intake diminished mRNA and protein of 11β-HSD2 by about 50% (p<0.001) and reduced the expression of the MR (p<0.01). The functionally relevant ENaC-β and ENaC-γ expression, a measure of mineralocorticoid action, diminished by more than 50% by high salt intake (p<0.001). The observed changes were present in rats with and without UNX. Thus, colonic epithelial cells appear to contribute to the protective armamentarium of the mammalian body against salt overload, a mechanism not modulated by UNX.

## Introduction

Sodium reabsorption is an established mechanism of the rectal and colonic mucosa [1], [2]. This mechanism is regulated, at least in part, by the mineralocorticoid receptor [3] with consecutive activation of the epithelial sodium channel (ENaC) [4]. Concomitantly with the increase in sodium reabsorption, colonic potassium excretion is enhanced by mineralocorticoids. Whether the latter effect is due to changes in the expression of some potassium channels or to modulation of the transepithelial voltage awaits clarification. Activation of the MR by fludrocortisone enhances the rectal electrical potential difference, an effect that is mimicked by inhibiting the enzyme 11β-HSD2 in segments of normal rectal colon obtained from humans [5]. The 11β-HSD2 enzyme converts endogenous cortisol into cortisone in humans and dehydrocorticosterone into corticosterone in rodents in mineralocorticoid target tissues including epithelial cells of the colon [6], [7]. This mechanism protects the MR from promiscuous activation by the glucocorticoids cortisol and corticosterone [8], [9], [10]. The clinical relevance of extra renal 11β-HSD2 has been demonstrated in patients without renal function [11], [12].

Sodium homeostasis depends on both, sodium intake and renal function. The response of the mammalian body to a high salt intake is an increased urinary loss of sodium, a teleologically sound response in order to maintain total body sodium balance. The molecular mechanism for this response is a down regulation of ENaC-β and ENaC-γ in the renal cortical collecting duct [13]. Here we hypothesized that a high salt intake diminishes these ENaC components also in the colon, a potentially useful mechanism to control intestinal sodium absorption. Whether a high salt intake modulates intestinal 11β-HSD2, a main determinant of ENaC expression is unknown and was therefore analyzed in the present investigation. In order to study the impact of a slightly diminished glomerular filtration rate on the molecular mechanisms of intestinal sodium handling the uninephrectomy (UNX) model was chosen. This model is of potential relevance for the increasing number of human living kidney donors. Na+ absorption by the rat distal colon is increased in the 5/6 nephrectomy model of chronic renal failure, an effect paralleled by an elevated expression of ENaC [14]. The purpose of the present investigation was to analyse the relevance of these observations in rats with moderate renal failure, i.e. after UNX.

Here we present evidence that high salt intake diminishes mRNA and protein levels of 11β-HSD2 and MR in colonic epithelial cells. Consistent with diminished MR action, the β- and γ-subunits of ENaC declined, a mechanism that contributes to the protection of the mammalian body from the deleterious effect of high salt intake. Fortunately, this protective mechanism also is present after UNX.

## Materials and Methods

### Animal experiments

All animal experiments were approved by the Ethical Committee of the Veterinary Administration of the Canton of Berne, Switzerland.

Male Sprague-Dawley rats (Charles River) were uni-nephrectomized (UNX) or sham operated (sham) at the age of 8 weeks. For surgical procedures, anesthesia was initiated with 450 µl/kg sc. of a mix containing 1.5 ml Dormitor (1 mg/ml, Pfizer, Karlsruhe, Germany), 2 ml Climasol (10 mg/ml, Gräub, Berne, Switzerland) and 1 ml Fentanyl (0.5 mg/ml, Janssen Cilag, Baar, Switzerland) and 100 µl/kg sc. were administrated for maintenance when needed. After deliberation of the right kidney from the perinephritic fat capsule, nephrectomy was performed by placing a double ligature around the renal artery, the renal vein and the ureter. In sham operated animals, the right kidney was exposed and the surgical site closed. Anesthesia was antagonized with 680 µl/kg sc. of a mix containing Antisedan (1 mg/ml, Pfizer, Karlsruhe, Germany), Sarmasol (1 mg/ml, Gräub, Berne, Switzerland), and Narcan (0.4 mg/ml, Opopharma, Zurich, Switzerland). Post-operative analgesia was controlled 2 times a day for 5 days with 0.9 mg/kg Temgesic (Redkitt, Benckiser, Switzerland) sc. After surgery rats had free access either to a regular chaw diet containing 0.4% NaCl or a high salt diet containing 8% NaCl (Provimi Kliba AG, Kaiseraugst, Switzerland) for 5 weeks. The composition of the rest of the food remained the same, while the potassium concentrations were equal for both diets One week before sacrifice; rats were housed individually in metabolic cages for 24 h urine collections. The 24 h urine was collected every day. For the analyses (results in Table 1) the mean of the last three days was considered. Rats were sacrificed with CO_2_, intestine was removed, placed in ice cold PBS and immediately processed as described below. Blood was obtained by puncturing the aorta abdominalis and collected in heparin tubes and plasma was obtained by centrifugation and stored at −20°C until use.

**Table 1:** Effect of uninephrectomy (UNX) and normal (NS) or high (HS) salt consumption on weight, plasma and urinary parameters.

		Sham NS n = 7	UNX NS n = 7	Sham HS n = 7	UNX HS n = 7
Weight	Body weight [g]	452±9	436±8	391±7^F^	385±7^F^
	Kidney [g]	1.94±0.18	2.25±0.06	2.73±0.12^E^	3.44±0.22A, F
Plasma	Na^+^[mmol/l]	129.6±1.4	132.4±2.6	136.6±1.6	136.6±1.4
	K^+^[mmol/l]	4.77±0.55	4.51±0.21	4.13±0.13	5.23±0.44
	Urea [mmol/l]	6.3±0.4	8.6±0.3	5.6±0.8	7.6±0.7
	Creatinine [µmol/l]	31±2	35±3	26±2	30±4
	Aldosterone [pg/ml]	424±103	431±130	283±106	309±94
	Corticosterone [ng/ml]	187±19	181±18	217±9	192±18
Urine	Volume [ml]	18.4±2.4	17.7±2.3	61.6±3.2^F^	73.3±2.6A, F
	Na^+^[mmol/24 h]	1.7±0.1	1.7±0.2	31.3±1.2^E^	29.1±0.5^D^
	K^+^[mmol/24 h]	2.71±0.6	1.77±0.15	1.81±0.07	1.51±0.03
	Cl^−^[mmol/24 h]	4.3±0.3	4.8±0.9	32.1±1.2^E^	29.1±0.6^D^
	GFR[ml/min]	3.0±0.3	2.3±0.1	3.7±0.4	2.4±0.1^A^

Values are given as Mean ± SEM. For statistical significance,

A p<0.01, Sham vs. UNX;

D p<0.01, NS vs. HS;

E p<0.001, NS vs. HS;

F p<0.0001, NS vs. HS.

### Intestine dissection

Rat whole intestines removed immediately after sacrifice were kept in cold PBS. Four sections were collected according to Sheppard et al. [15]: duodenum, 10 cm distal to the pylorus; ileum, 10 cm proximal to the cecum; proximal and distal half of the colon. Tissue sections opened longitudinally and washed with cold PBS were decontaminated by soaking in 0.04% sodium hypochlorite (Sigma-Aldrich, St. Louis, MO, USA) in PBS for 30 min on ice and washed in PBS.

### Epithelial cell isolation

Cells were prepared from intestinal sections by a non-enzymatic technique according to Whitehead et al. [16], [17]. After decontamination tissue sections were cut into small pieces (length ∼2 cm) and incubated in 50 ml Falcon tubes containing a solution of 1 mM EDTA, 1 mM EGTA, 0.5 mM DTT in PBS, with occasional gentle shaking at room temperature (RT). The solution was replaced by cold PBS after 1.5 h. Cells were detached by shaking the tube vigorously 10 times by hand. This shaking procedure was repeated three times using cold PBS. The pooled cells were centrifuged at 179×g for 5 min at 4°C and pellets kept frozen at −70°C.

### RNA isolation, reverse-transcription and real-time PCR (TaqMan)

Total RNA was extracted with Trizol Reagent (Invitrogen, Carlsbad, CA, USA) from frozen colon epithelial cells, the concentration of which was determined spectroscopically (Nano Drop 1000 spectrometer, NanoDrop products, Wilmington, DE, USA). One µg of RNA was reversed-transcribed with ImProm-II reverse transcriptase (Promega, Madison, WI, USA) using 0.25 µg Oligo-dT primers (Invitrogen), 0.5 µl (∼1 µg) random hexamers (Roche, Indianapolis, IN, USA) and 2.4 mM MgCl_2_ in a volume of 25 µl according to the manufacturer's protocol. Reactions containing cDNA (30 ng), TaqMan primer/probe mix (Applied Biosystems, Carlsbad, CA, USA) and TaqMan Fast Universal Master Mix (No AmpErase UNG, Applied Biosystems) were analyzed by real-time PCR using the 7500 Fast Real-Time PCR System (Applied Biosystems). β-actin and GAPDH were used as internal controls. The relative gene expression was calculated using the 2(-ddCt) method [18]. The following TaqMan rat gene expression assays (dye FAM) were used: 11β-HSD2: Rn00492539_m1; MR (gene Nr3c2): Rn00565562_m1; GR (gene Nr3c1): Rn00561369_m1; ENaC-α, -ß, -γ (genes Scnn1-α, -ß, -γ): Rn00580652_m1, Rn00561892_m1, Rn00566891_m1; Sgk-1: Rn00570285_m1; GILZ (gene Tsc22d3): Rn00580222_m1; TaqMan endogenous controls (dye VIC, primer limited): β-actin: 4352340E and GAPDH: 4352338E.

### Western blot analysis

Cells were homogenized by sonification in sucrose buffer (250 mM sucrose, 10 mM Tris-HCl, pH 7.5) containing protease inhibitors (Mini Protease Inhibitor Cocktail Tablets, Roche). Lysates were centrifuged at 3'000 rpm for 10 min at 4°C and supernatants frozen at −70°C. Protein concentrations were determined by Pierce BCA protein assay (Thermo Scientific, Rockford, IL, USA). 50 µg of protein were separated using 8% SDS-PAGE in running buffer (192 mM glycine, 25 mM Tris base, 0.1% SDS) at 100 V for 2 h. Proteins were blotted to nitrocellulose or PVDF membranes (Hybond-C/Hybond-P, GE Healthcare Life Sciences, Piscataway, NJ, USA) in transfer buffer (192 mM glycine, 25 mM Tris base, 20% methanol) on ice for 2 h at 2.3 mA/cm. After blocking with 5% dry milk in TBS-T (137 mM NaCl, 20 mM Tris, 0.1% Tween 20, pH 7.6), membranes were incubated with primary and secondary antibodies, each followed by three wash periods in TBS-T of 15 min. For GAPDH detection TopBlock (Lubio Science, Lucerne, Switzerland) was used in addition to milk throughout the protocol. For signal detection, membranes were incubated in ECL™ Western Blotting Detection Reagents (GE Healthcare) and exposed to X-ray films (Hyperfilm ECL, GE Healthcare). Band intensities were quantified with ImageJ (http://rsb.info.nih.gov/ij/). Protein amounts were normalized with endogenous actin and GAPDH. The following antibodies were used (Santa Cruz Biotechnology, Santa Cruz, CA, USA): Primary antibodies: sheep anti-rat 11β-HSD2 (AB1296, Millipore (Chemicon), Billerica, MA, USA), 1∶3'000; rabbit anti-MR (sc-11412), 1∶500; rabbit anti-GR (H-300, sc-8992), 1∶500; rabbit anti-actin (sc-1616-R), 1∶20'000; goat anti-GAPDH (sc-20357), 1∶1'000; Secondary antibodies: Goat anti-rabbit IgG-HRP (sc-2004), 1∶10'000 (for actin 1∶20'000); rabbit anti-sheep IgG-HRP (sc-2770), 1∶10'000; donkey anti-goat IgG-HRP (sc-2304), 1∶10'000.

### 11β-HSD2 activity assay

Since the activity assessed in extracted epithelial cells was not reliably detectable, pieces of 0.5–1 cm^2^ size were cut from decontaminated rat intestine. The tissue pieces were placed into Eppendorf tubes containing 250 µl of HBSS (Gibco 14025) with 25 nM ^3^H-corticosterone (78.5 Ci/mmol, Perkin Elmer, Waltham, MA, USA) and 0.1 mM NAD^+^. Reactions were incubated at 37°C and stopped after 30 min by cooling on ice and adding 250 µl of stop solution (corticosterone and 11-dehydrocorticosterone in methanol, 2 mg/ml each). Metabolites were separated on silica plates (UV_254_, Macherey-Nagel, Oensingen, Switzerland) by thin-layer chromatography using chloroform/methanol 9∶1. Steroids were transferred to scintillation vials and radioactivity was measured in a β-counter (Tri-Carb 2000CA, Canberra Packard) using 4 ml of scintillation solution (Irgasafe, Zinsser Analytic, Frankfurt, Germany).

### Measurement of corticosterone and aldosterone in plasma

Corticosterone was analyzed in our laboratory by gas chromatography-mass spectrometry [19] and aldosterone by a commercially available ELISA Kit (Uscn Life Science Inc., Wuhan, China).

### Assessement of clinical chemistry parameters and calculation of renal function

Creatinine, chloride, urea, potassium, and sodium levels were determined in serum and urine by routine methods and the GFR was calculated by considering serum and urinary creatinine levels (Table 1).

### Statistics

Graphs and statistical analysis were realized with Graphpad Prism version 5.01 (GraphPad Software, San Diego, California, USA). Each rat group (n = 7) was tested for normal distribution with the Shapiro-Wilk normality test. To analyze impact of salt intake and UNX between the rat groups two-way ANOVA and Bonferroni's post-hoc test were applied. Enzyme activities between intestine segments were analyzed by Friedman and Dunn's post test. The impact of high salt and UNX on activity was analyzed by Kruskal-Wallis and Dunn's post test for each intestine part. Values in Table 1 were assessed by one-way Anova and Bonferroni's post-oc test or by Kruskal-Wallis followed by Dunn's multiple comparison test (plasma: potassium, urin: sodium, potassium, chloride).

The significance level alpha was set to 0.05.

## Results

### Effect of UNX, normal or high salt consumption on weight, plasma and urinary parameters

The body weight of sham and UNX rats fed with a high salt diet was significantly lower compared to that of rats on a normal salt diet (Table 1). The kidney weight after UNX rat was slightly higher. The aldosterone concentrations displayed a large variation with a tendency to lower values on high salt in both groups of animals, sham and UNX. As expected sodium and chloride excretion increased in high salt treated animals while potassium remained unchanged. Renal function appeared by and large to be slightly reduced in UNX when compared with sham operated rats, as evidenced by the difference in serum urea concentrations and the differences in GFR (Table 1).

### Differential activity of 11β-HSD2 within rat intestine and impact of UNX and salt intake

**Figure 1:**
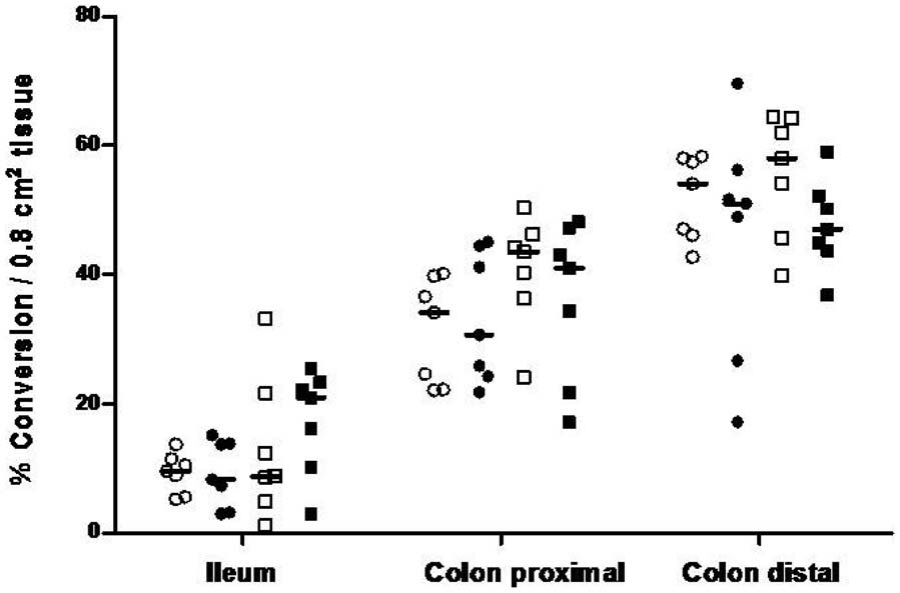
11β-hydroxysteroid dehydrogenase type 2 activity in rat intestine. Enzyme activity in total tissue was assessed by incubating fresh tissue sections in the presence of ^3^H-labelled substrate corticosterone. Metabolites were separated by thin-layer chromatography. Conversion rates of ^3^H-corticosterone (B) into ^3^H-11-dehydrocorticosterone (A) were calculated as percentage of dpm A/(dpm A+dpm B) and expressed per area of tissue. Sham normal salt: open circles, UNX normal salt: closed circles, sham high salt: open squares, UNX high salt: closed squares. The median for each group is given by a horizontal line (n = 7).

Activity measurements of 11β-HSD2 were performed in tissue sections obtained from three different parts of rat intestine (Figure 1). Results confirmed data from previous studies, suggesting intestinal segment specific 11β-HSD2 activities (p<0.001) [15], [20], [21]. Conversion of corticosterone to 11-dehydrocorticosterone increased significantly from small intestine to colon and was highest at the end of the colon (ileum vs. colon proximal: p<0.001, colon proximal vs. colon distal: p<0.05). The variability of the enzyme activities between the groups was comparable except two UNX animals on high salt diet and two control animals on normal salt diet had very low activities in the colon proximal and distal, respectively. Considering the enzyme activity in total tissue of ileum, proximal and distal colon, there was no significant effect of UNX and/or salt intake.

### 11β-HSD2 is down-regulated in colon epithelium by high salt intake but unaffected by UNX

**Figure 2:**
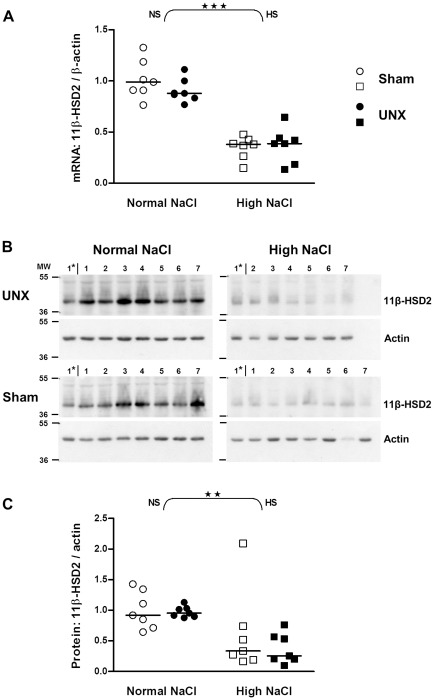
Impact of salt intake on 11β-hydroxysteroid dehydrogenase type 2 expression in the colon of control and UNX rats. A: mRNA expression in epithelial cells isolated from colon (real-time PCR). Values were normalized with endogenous β-actin mRNA expression. Results were quantified relative to the mean of the sham-operated, normal salt group. B: Western blots showing protein in epithelial cells isolated from colon. Lanes 1–7: Rat colon samples; Lane 1*: Sample 1 of UNX high NaCl group was loaded on each gel as a standard for comparison between blots; MW: molecular weight. C: Amount of proteins relative to control rats (sham-operated, normal salt). Values were adjusted with the colon protein sample on lane 1* and normalized with the protein expression of actin. The horizontal line is the median (n = 7). ★★, P≤0.01; ★★★, P≤0.001 indicate the effect of salt analyzed by two-way ANOVA (curly brackets; NS: normal salt; HS: high salt).

Since 11β-HSD2 is located in the sodium-transporting epithelia of the intestinal tract epithelial cells were isolated from underlying tissue by a non-enzymatic technique [16], [17]. When compared with normal salt, high salt diet decreased 11β-HSD2 mRNA expression levels by 65% and 59% in control and UNX rats, respectively (for both p<0.001; Figure 2A). In line with the reduced mRNA expression, high salt diet lowered 11β-HSD2 protein in colon epithelium (p<0.01; Figure 2C). While the overall effect of salt was significant for both mRNA and protein levels, UNX had no effect on 11β-HSD2 (Figure 2).

### High salt diet decreases expression of MR, but not GR in colon epithelium

**Figure 3:**
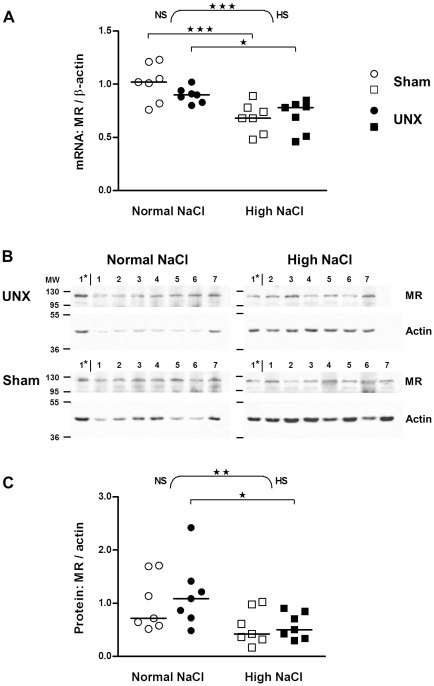
Impact of salt intake on mineralocorticoid receptor expression in the colon of control and UNX rats. A: mRNA expression in epithelial cells isolated from colon (real-time PCR). Values were normalized with endogenous β-actin mRNA expression. Results were quantified relative to the mean of the sham-operated, normal salt group. B: Western blots showing protein in epithelial cells isolated from colon. MR detection was done on the same membranes used for GR before (Figure 4). For reprobing membranes were incubated in stripping buffer (2% SDS, 62.5 mM Tris-HCl pH 6.8, 0.75% beta-mercaptoethanol in H_2_O) for 30 min at 50°C while shaking and washed three times in TBS-T. Lanes 1–7: Rat colon samples; Lane 1*: Sample 1 of UNX high NaCl group was loaded on each gel as a standard for comparison between blots; MW: molecular weight. C: Amount of proteins relative to control rats (sham-operated, normal salt). Bands quantified as in Figure 2. The horizontal line is the median (n = 7). ★, P≤0.05; ★★, P≤0.01; ★★★, P≤0.001 indicate the effect of salt by two-way ANOVA (curly brackets; NS: normal salt; HS: high salt) or significant differences between normal and high salt groups (squared brackets, Bonferroni's post test).

A high salt intake diminished both mRNA and protein levels of the MR (mRNA: p<0.001, protein: p<0.01; two-way ANOVA; Figure 3). Mean values were diminished by 33% (p<0.001) and 22% (p<0.05) for mRNA in sham operated and UNX rats, respectively (Figure 3A) and by 44%, (non-significant, p>0.05) and 51% (p<0.05) for protein in sham operated and UNX rats, respectively (Figure 3C).

**Figure 4:**
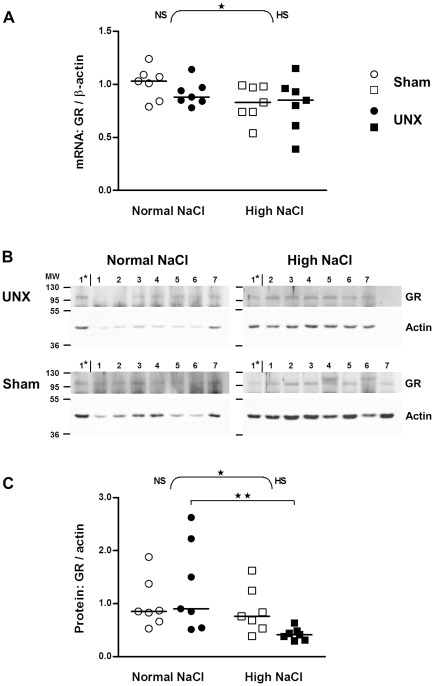
Impact of salt intake on glucocorticoid receptor expression in the colon of control and UNX rats. A: mRNA expression in epithelial cells isolated from colon (real-time PCR). Values were normalized with endogenous β-actin mRNA expression. Results were quantified relative to the mean of the sham-operated, normal salt group. B: Western blots showing protein in epithelial cells isolated from colon. Lanes 1–7: Rat colon samples; Lane 1*: Sample 1 of UNX high NaCl group was loaded on each gel as a standard for comparison between blots; MW: molecular weight. C: Amount of proteins relative to control rats (sham-operated, normal salt). Bands quantified as in Figure 2. The horizontal line is the median (n = 7). ★, P≤0.05 indicates the effect of salt analyzed by two-way ANOVA (curly brackets; NS: normal salt; HS: high salt). ★★, P≤0.01 indicates a significant difference between the UNX normal salt and UNX high salt group (squared bracket, Bonferroni's post test).

The GR mRNA appeared to decline only slightly after high salt intake when normalized with β-actin (p<0.05 for effect of salt by two-way ANOVA) but not when normalized with GAPDH. High salt intake had no effect on mRNA expression of GR within the control and UNX rat groups (Bonferroni's post test, Figure 4A). In line with the tendency of reduced mRNA expression of GR, high salt intake decreased the protein levels of GR when control and UNX rats were analyzed as one group (p<0.05, two-way ANOVA), an effect only significant in UNX (p<0.01) but not in control rats (Figure 4C).

### High salt diet decreases colonic epithelial sodium channel (ENaC) subunits

To explore the molecular effects of the observed changes in 11β-HSD2 and MR several genes known to be induced upon stimulation with glucocorticoids were investigated. For this purpose mRNA transcripts of the ENaC subunits α, β, γ, serum/glucocorticoid regulated kinase 1 (Sgk-1) and glucocorticoid-induced leucine zipper protein (GILZ) were analyzed in colon epithelial cells.

**Figure 5:**
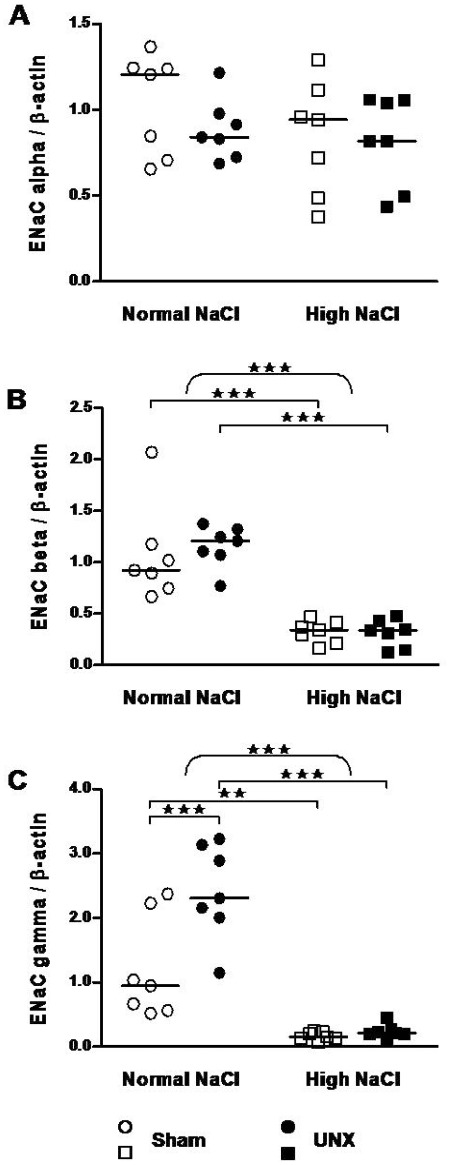
Impact of salt intake and uni-nephrectomy on epithelial sodium channel (subunits α, β, γ) mRNA expression in the rat colon. mRNA expression in epithelial cells isolated from colon (real-time PCR). Values were normalized with endogenous β-actin mRNA expression. Results were quantified relative to the mean of the sham-operated, normal salt group. Epithelial sodium channel subunits: A, alpha, B, beta and C, gamma. The horizontal line represents the median (n = 7). ★★, P≤0.01; ★★★, P≤0.001 indicate the effect of salt by two-way ANOVA (curly brackets; NS: normal salt; HS: high salt) or significant differences between groups (squared brackets, Bonferroni's post test).

Whereas the alpha subunit of ENaC remained unchanged (Figure 5A), high salt intake strongly reduced expression of the beta subunit of ENaC by 3.7-fold in control and 3.3-fold in UNX rats, respectively (for both p<0.001; Figure 5B). The reduction of the gamma subunit of ENaC was even more pronounced by 7.2-fold (p<0.01) and 10-fold (p<0.001) in control and UNX rats, respectively (Figure 5C). A two-way ANOVA revealed besides the effect of salt (p<0.001, curly bracket) a significant effect of UNX (p<0.01, not indicated) as well as a significant salt×UNX interaction (p<0.01, not indicated). In particular the expression of ENaC-γ was enhanced 2-fold by UNX in rats with normal diet (p<0.001) but not with high salt diet (Figure 5C).

Sgk-1 tended to decline with high salt intake. A two-way ANOVA showed a significant effect of salt when results were normalized with GAPDH (p<0.05) but not with β-actin (p>0.05; data not given). In opposite to Sgk-1, GILZ was slightly increased by high salt intake (p<0.01 by two-way ANOVA). This effect was only significant between the UNX groups (p<0.05, data not shown).

## Discussion

The maintenance of sodium and potassium homeostasis requires a complex interplay between nutritional input and excretion of these electrolytes. Besides the kidneys the gastrointestinal tract plays a central role for the absorption and excretion of sodium and potassium. Therefore the analysis of the regulation of the key elements responsible for intestinal electrolyte handling is of interest. Here we report that first, the 11β-HSD2 is segment specifically expressed along the entire colon when whole tissue was analyzed with the highest activity in the distal colon (Figure 1), second, a high salt diet down-regulates the expression of 11β-HSD2, MR and ENaC-β- and -γ components and third, UNX does not abrogate or enhance the effect of a high salt diet. Our finding might have some limitations since analyzing total tissue we did not take into consideration a possible inconsistent content of epithelial tissue in these segments. Most likely for some technical reasons we were not able to detect 11β-HSD2 activity in isolated epithelial cells, however mRNA and protein levels were assessed without difficulty. When comparing our regional distribution patterns with those of other groups they were in line [15], [20]. Similar to our results Pácha and Mikšík noticed higher conversion rates in the distal than in the proximal colon [20] an observation contrasting that of Sheppard et al reporting similar conversion rates in both segments [15].

The impact of salt intake and corticosteroids on the activity of 11β-HSD2 in defined intestinal segments is controversial. A low salt intake increased and decreased the activity in proximal colon and ileum respectivly, a finding attributed to increased aldosterone concentrations, whereas abrogation of aldosterone/corticosterone production by adrenalectomy increased the activity in both, ileum and proximal colon [20]. In vitro studies with explants of rat ileum and colon revealed induced activity in the presence of aldosterone or dexamethasone, an effect blocked by the 11β-HSD inhibitor carbenoxolone [21]. In the present study, which investigated for the first time UNX rats on a normal and high salt diet the activity of 11β-HSD2 did not change when assessed in total fresh tissue sections (Figure 1), a finding suggesting that UNX and/or a high salt diet are not involved in the regulation of the 11β-HSD2 enzyme activity. This conclusion is not completely correct for the following reasons: In addition to 11β-HSD2 expression in mucosal epithelial cells, the bidirectional 11β-HSD1 is expressed in cells of the lamina propria [22]. The 11β-HSD1 oxidizes corticosterone to 11-dehydrocorticosterone or cortisol to cortisone under the conditions as used for the assessment of the 11β-HSD2 activity in cell lysates [23]. The presence of both 11β-HSD isoforms in the whole colon, located in distinct tissue compartments [22], might therefore be a confounding variable and an obstacle for measuring 11β-HSD2 activity specifically.

In order to improve the sensitivity and specificity of the measurements we performed in addition enzyme activity determinations in protein lysates of colon tissue adding 11β-HSD2 specific cofactors. In line with previous measurements in homogenized colonic tissue [24], virtually no activity of 11β-HSD2 was detectable in these lysates (results not given). We tried also to measure enzyme activity in homogenates from frozen epithelial cells from colon according to Sheppard et al [15]. The conversion rate however was very low and inconsistent. Using higher amounts of protein and/or longer incubation periods did not improve the results. Given these methodological uncertainties we focused on fresh tissue sections and isolated intestinal epithelial cell.

Similar to enzyme activity, levels of mRNA and protein of 11β-HSD2 were much more abundant in colon than in the small intestine [15], [25] Given the known anatomical distribution we expected a higher expression of 11β-HSD2 in epithelial cells with negligible amounts of 11β-HSD1 when compared with that of total colon tissue [15]. The composition of epithelial cells harvested depends on the incubation time with the chelating agent. Cells were detached along the crypt axis in apical to basal direction, thus surface epithelial cells were liberated earlier than cells at the crypt base [22]. Due to its subcellular localization, 11β-HSD2 activity was present in both nuclear and microsomal pellets of human and rat colon, but not in cytosol when no detergent was added [26], [27]. These technical details, well standardized for the present investigation may explain at least in part the discrepancies observed between our results and the results reported for intestinal 11β-HSD2.

The standardization of intestinal mRNA values is of concern since the internal standard might change by the intervention. Therefore we normalized mRNA values with two different standards, β-actin and GAPDH expression. Similar results were obtained with both internal standards. High salt diet clearly decreased 11β-HSD2 mRNA expression and protein in epithelium isolated from the entire colon, while UNX did not modulate the enzyme expression (Figure 2). Rats were fed a normal and a high salt diet. While the rats were on a high salt diet the serum aldosterone concentrations declined slightly, albeit not significantly (Tab. 1). Since aldosterone has been shown to increase 11β-HSD2 expression [28] it is conceivable that the changes in aldosterone concentration accounted at least in part for the changes in 11β-HSD2 expression.

11β-HSD2 is crucial for controlling MR action since a reduced 11β-HSD2 expression (more than 2-fold, Figure 2) causes an increased intracellular availability of 11β-hydroxy-glucocorticoids for the MR [8], [10], [29]. Thus we explored the effect of UNX or high NaCl diet on MR and GR expression. UNX did not modulate MR and GR expression, whereas a high salt diet caused a down-regulation of mRNA and protein of the MR. The absence of an effect of high salt intake on the GR is in line with observations made by Nørregaard et al [30]. However, Nørregaard et al found increased MR mRNA in total colon tissue samples from rats on a high NaCl diet [30], while we observed a decrease in mRNA and protein of the MR in isolated epithelial cells. The difference is probably best explained by the difference in the biological materials considered. The observed down-regulation of MR expression might compensate for the effect of the down-regulated 11β-HSD2 with respect to the overall mineralocorticoid action. Therefore a biological effect representative for the mineralocorticoid action has to be considered.

In rat colon aldosterone induces an amiloride-sensitive short-circuit current, an effect that is primarily attributed to sodium transport by ENaC in the epithelium [31]. Mechanisms mediating this effect include transcriptional regulation of ENaC through activation of MR [32], and ubiquitination of ENaC subunits by Nedd4-2 controlling channel stability [33]. This process is counteracted by Sgk-1, which phosphorylates Nedd4-2 thereby preventing its interaction with ENaC [34]. Another important factor for ENaC regulation is GILZ, which prolongues the half-life of Sgk-1 by inhibiting its degradation [35]. In the present study the mRNA transcripts of the mineralocorticoid-induced genes ENaC (α, β, γ), Sgk-1 and GILZ were analyzed. We observed an unchanged ENaC-α expression in the presence of a remarkable decrease of ENaC-β and -γ after the high NaCl diet when compared with the normal NaCl diet (Figure 5). The differential response of the ENaC-α vs. ENaC-β and ENaC-γ is in line with the literature [36], [37], [38]. In the distal colon from rats, low dietary NaCl or high concentrations of mineralocorticoids increased the β- and γ-subunits whereas the α-subunit remained unchanged [38], [39], [40], [41]. Here we did not detect a change in Sgk-1 expression as a function of salt intake. In our study rats were fed a high salt diet for five weeks, thus not unexpectedly no changes in Sgk1-expression were observed, since Sgk-1 is an immediate early gene, the expression of which increases to a steady-state within hours [42]. The results about Sgk-1 from the literature are ambiguous. Sgk-1 was decreased after adrenalectomy in the colon, but remained unchanged after sodium depletion or aldosterone injection [39], while other studies reported an induction by these interventions [43], [44] Unexpectedly, GILZ expression exhibited a tendency to increase rather than decrease under high NaCl diet (data not shown). We postulate that GILZ may be regulated in the opposite direction of 11β-HSD2 and ENaC and behaves like the sodium-hydrogen exchanger NHE-3 mRNA and protein [45]. The reduced NHE-3 expression with low NaCl was normalized when carbenoxolone was given. The authors concluded that probably an enhanced 11β-HSD2 activity suppressed the glucocorticoid-GR pathways during NaCl restriction in colon [30].

Taken together, we report that high salt intake causes two functionally opposing effects, namely a decline in the expression of 11β-HSD2 and MR. A decline in the expression of 11β-HSD2 favors the access of cortisol to the MR and therefore enhances mineralocorticoid action, whereas a diminished expression of the MR reduces mineralocorticoid action. Based on the diminished expression of the two aldosterone-dependent proteins, ENaC-β and ENaC-γ it is reasonable to conclude that salt induced inhibition of MR expression is functionally dominant in epithelial cells from the colon. Teleological these observations may be interpreted as an intestinal contribution to the protective armamentarium of the mammalian body against high salt intake. It appears that the protective mechanism of high salt is not abrogated by UNX. Further studies are needed to establish whether these findings are also present in humans.
